# Perforated duodenal ulcer in the third trimester of pregnancy, with survival of both the mother and neonate, in Ethiopia: a case report

**DOI:** 10.1186/s13256-022-03562-w

**Published:** 2022-08-29

**Authors:** Tafese Dejene Jidha, Keno Mohammed Umer, Girma Beressa, Tadesse Tolossa

**Affiliations:** 1 College of Medicine and Health Sciences, Dire Dawa University, Dire Dawa, Ethiopia; 2 Department of Public Health, Goba Referral Hospital, Madda Walabu University, Bale Goba, Ethiopia; 3 Department of Public Health, Institute of Health Science, Wollega University, Nekemte, Ethiopia

**Keywords:** Perforated peptic ulcer disease, Third-trimester pregnancy, Survive, Laparotomy

## Abstract

**Background:**

Perforated peptic ulcer disease is a serious complication of peptic ulcer disease (PUD) that presents as acute abdomen. It is very uncommon during pregnancy, but its diagnosis in pregnancy is very challenging in general, and more so in the third trimester. Timely diagnosis and prompt surgical intervention can prevent maternal and fetal mortality, but delayed diagnosis is linked with poor maternal and fetal outcomes. The aim of this case report is to emphasize the need for healthcare professionals to consider the differential diagnosis of perforated PUD when presented with cases of acute abdomen in pregnancy and to involve a multidisciplinary team in management for better feto-maternal outcome.

**Case presentation:**

A 35-year-old pregnant Ethiopian woman, Gravida 7 and Para 6, presented with a sudden onset of right upper quadrant pain, nausea, and vomiting of 7 hours duration at 36 weeks of gestation. She also had contractions and leakage of liquor of two hours duration. Her abdomen was grossly distended, rigid, and diffusely tender, and showed limited movement with respiration. An upright abdominal X-ray demonstrated air under the diaphragm. She was diagnosed with perforated peptic ulcer disease. Labor was augmented, and a 2.9-kg live male neonate was delivered vaginally. Two hours after delivery, laparoscopic omental patch repair was performed. The patient was discharged 7 days after the omental patch repair surgery in stable condition.

**Conclusions:**

Perforated PUD in pregnancy is a rare occurrence, which may account for the delay in diagnosis and management. Obstetricians should keep a high index of suspicion when a pregnant woman presents with acute abdomen. Care provided by obstetricians should be coupled with care provided by other disciplinary teams, in order to reduce maternal and fetal morbidity and mortality.

## Background

Peptic ulcer disease (PUD) and its complications, including perforated peptic ulcer, during pregnancy are very uncommon, ranging from one to six in every 23,000 pregnancies [1–3]. Peptic ulcers are believed to heal during pregnancy secondary to protective physiological changes during pregnancy and maternal avoidance of ulcerogenic factors, such as cigarette smoking and alcohol intake [4]. Life-threatening complications of PUD are perforation and bleeding. Perforated PUD is the commonest cause of peritonitis in the general population, but it rarely occurs in pregnancy. It presents as acute abdomen, but its diagnosis is very challenging in pregnancy in general and more so in the third trimester. Factors contributing to the delayed diagnosis of perforated PUD include the rarity of the disease in pregnancy and non-specific symptoms of the disease. Due to fears for fetal safety, the use of diagnostic radiography modalities, such as plain abdominal X-ray or computed tomography (CT) scans, to establish the diagnosis of perforated PUD is limited. Timely diagnosis and prompt surgical intervention can prevent maternal and fetal mortality [1, 3], but delayed diagnosis is linked with poor maternal and fetal outcomes. To our knowledge, there are no case reports or case series that show survival of mothers and neonates from perforated PUD during the third trimester of pregnancy in resource-limited settings like Ethiopia. Here, we report the case of perforated PUD in a pregnant woman in Ethiopia, with survival of both the mother and neonate. This case report is crucial in that it emphasizes the need for healthcare professionals to keep a differential diagnosis of perforated PUD in mind when dealing with cases of acute abdomen in pregnancy and to involve multidisciplinary team in management for better feto-maternal outcome.

## Case presentation

A 35-year-old Ethiopian woman, Gravida 7 and Para 6, with no significant past medical history presented to Dilchora Referral Hospital at gestational age of 36 weeks with a sudden onset right upper quadrant pain of 7 hours duration that radiated to her back. She had experienced nausea and vomiting during the night prior to her presentation at the hospital coupled with inability to tolerate oral intake. Her presentation at the hospital was driven by worsening of these symptoms. She had history of intermittent burning type of epigastric pain prior to pregnancy. She also had contraction and leakage of liquor of 2 hours duration which started while on the way to our hospital. There was no history of vaginal bleeding.

**Fig. 1:**
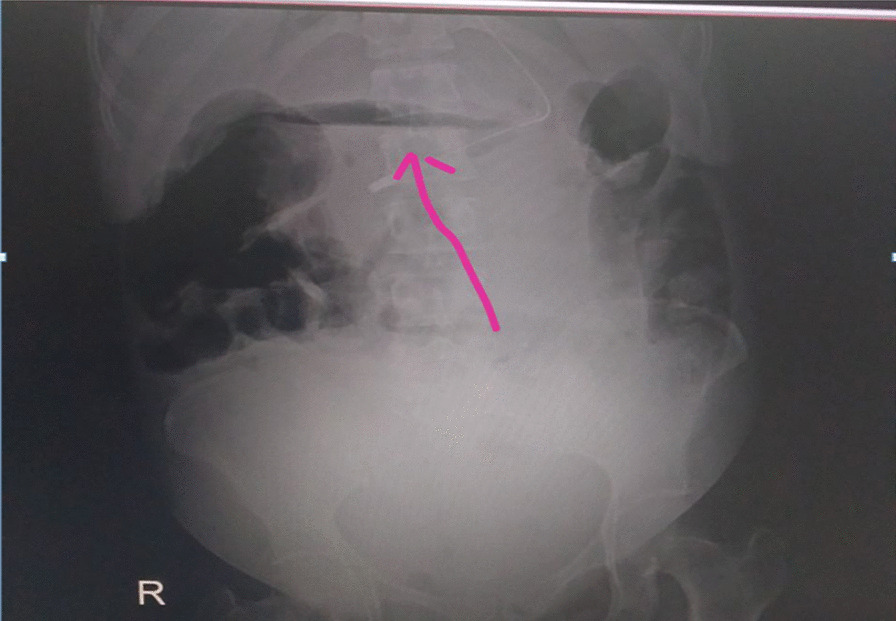
Upper abdominal X-ray taken before laparotomy showed air under the diaphragm (arrow)

Upon arrival, the patient was in acute distress, with the following vital signs: blood pressure, 95/60 mmHg; pulse rate, 132 beats per minute (bpm); respiratory rate, 32 breaths per minute; and body temperature, 35.9 °C. Abdominal examination showed a grossly distended, rigid, and diffusely tender abdomen, which showed limited movement with respiration. Uterine fundal height was consistent with the gestational age. There were three uterine contractions in 10 minutes, each lasting for 35–40 seconds. The fetal heart beat (FHB) ranged from 120 to 158 bpm. Digital vaginal examination revealed a cervix of 3-cm dilation, a ruptured membrane, clear liquor, and high station. An upright abdominal X-ray demonstrated air under the diaphragm (Fig. 1). A bedside ultrasound showed a single intrauterine pregnancy, positive FHB, fundal anterior placenta with a central thickness of 35 mm, gestational age of 36 weeks, an estimated fetal weight of 3000 g, no gross anomaly, and massive amount of intraperitoneal fluid.

**Fig. 2:**
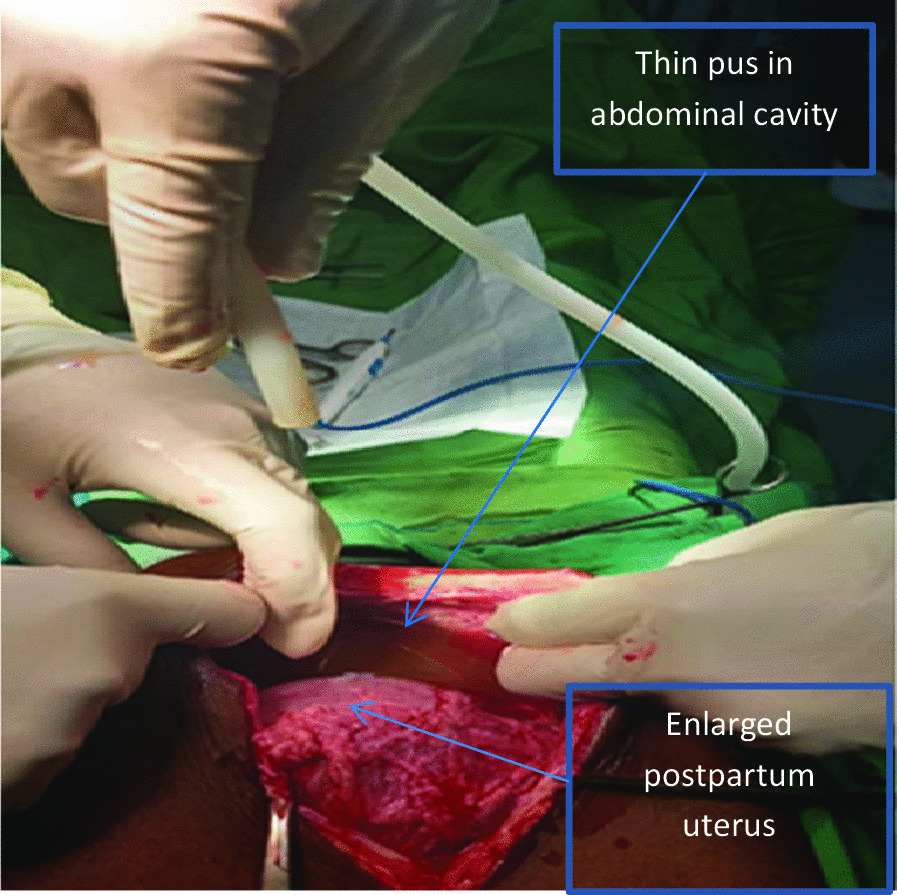
A copious amount of thin pus was found the abdominal cavity (arrow), which was suctioned out

**Fig. 3:**
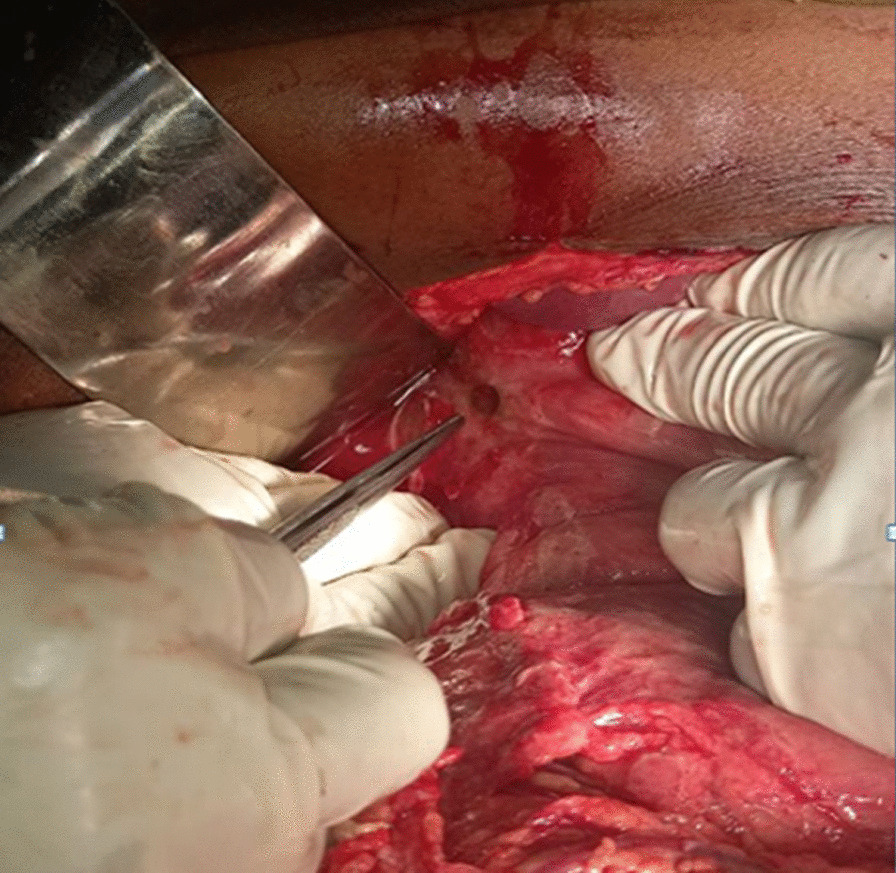
Artery forceps showing 0.5 × 0.5-cm anterior perforation on the first part of the duodenum intraoperatively

With the diagnosis of latent first stage of labor plus acute abdomen secondary to presumed perforated PUD, a general surgeon was consulted, and it was decided to perform laparotomy after delivery. She was resuscitated with crystalloids and put on intranasal oxygen, a nasogastric tube was inserted, and she was catheterized. She was started on intravenous ceftriaxone, metronidazole, and omeprazole. At 4 hours after admission, the labor was augmented with oxytocin, and an alive male neonate weighing 2900 g with an Apgar score of 5 and 8 in the first and fifth minutes respectively, was delivered vaginally. At 2 hours after delivery, a general surgeon performed a laparotomy via midline abdominal incision. This revealed a copious amount of thin pus in the abdominal cavity and a 0.5 × 0.5-cm anterior perforation of the first part of the duodenum (Figs. 2, 3). The perforation was repaired, and an omental patch (Graham’s patch) repair was performed. The patient was transferred to an intensive care unit (ICU) and was discharged 1 week later in stable condition with a prescription for 20 mg omeprazole daily for 1 month.

## Discussion

This case report describes a case of perforated PUD during pregnancy in which the both mother and newborn survive. Perforated PUD in pregnancy is an extremely rare complication. When it does occur, survival of both the mother and newborn is unusual. Early diagnosis and surgical management offer the best hope for the survival of mother and newborn.

The incidence of PUD during pregnancy decreases [2], and several theories have been put forward to explain this decrease. The rarity of peptic ulcer and of its complications in pregnancy correlate with the hypochlorhydria found in pregnant women and with increased secretions of the anterior pituitary-like hormones in the urine [5]. An increase in plasma histamine in pregnancy (caused by placental histaminase synthesis) increases metabolism of maternal histamine, thereby reducing gastric acid secretion during pregnancy [6]. It has also been suggested that female gestational hormones (particularly progesterone) decrease the rate of ulcer formation by increasing gastric mucus synthesis. Avoidance of ulcerogenic factors, such as alcohol, cigarette smoking, and nonsteroidal anti-inflammatory drugs (NSAIDS), all likely contribute to the reduced incidence of PUD in pregnancy.

Cardinal symptoms of patients with PUD are pain, nausea, and vomiting. The pain is often epigastric and worse at night. In the presence of a gravid uterus (especially when labor ensues), it might be quite difficult for patients to localize the pain. Perforated PUD presents as an acute abdomen, and its diagnosis can be very challenging, and possibly delayed, especially in women in their third trimester. Factors contributing to the delayed diagnosis of perforated PUD include rarity of the disease in pregnancy and the non-specific symptoms of the disease. The symptoms are mimicked by other common gastrointestinal problems in pregnancy, such as nausea and vomiting during pregnancy, hyperemesis gravidarum, gastroesophageal reflux disease, and cholecystitis. In our patient, the pain was sudden in the onset right upper quadrant pain that radiates to the back. She also had nausea and vomiting on the night prior to arrival at the hospital with an inability to tolerate oral intake.

Uncomplicated PUD presents with minimal physical signs, but when complicated with perforation, it presents with signs of peritonitis, including tachycardia, abdominal tenderness (or even guarding), and rebound tenderness. At presentation, our patient was in acute distress, with a pulse rate of 132 bpm and a respiratory rate of 32 breaths per minute; abdominal examination revealed a grossly distended, rigid, and diffusely tender abdomen that limited respiration movement.

In pregnancy, the use of diagnostic radiography modalities, such as an upright abdominal X-ray to establish the diagnosis of perforated PUD, are limited due to the potential deleterious effects of ionizing radiation on fetal safety [7]. However, such examinations must be performed when there is suspicion of perforated PUD in order to assess the presence of pneumoperitoneum: the maternal and fetal benefits of prompt diagnosis and treatment far outweigh any fetal risks of teratogenicity or childhood cancer. In our patient, an upright abdominal X-ray was performed and demonstrated air under the diaphragm, which supported the diagnosis of perforated PUD. Abdominal ultrasound evaluation is also useful as it can identify the indirect findings of perforation, such as decreased peristalsis and the presence of free fluid between bowel loops. In our patient, an abdominal ultrasound examination showed the presence of a massive amount of intraperitoneal fluid, which again supported the diagnosis the perforated PUD.

Management of perforated PUD in pregnancy requires a multidisciplinary team that includes obstetricians, surgeons, gastroenterologists and pediatricians. The management includes initial resuscitation with crystalloids, correction of electrolyte imbalance, nasogastric suction, administration of intravenous broad-spectrum antibiotics, and medications for PUD, such as proton pump inhibitors, followed by laparotomy after patient stabilization. The recommended surgery for perforated PUD is omental patch (Graham’s patch) repair which involves primary closure with the placement of an omental patch for support [2]. Non-obstetric surgery in pregnancy increases the incidence of obstetrics complications, such as preterm labor [8]. Hence, steroid administration to enhance lung maturity should be considered in patients who are at risk for surgical intervention at preterm gestation. Our patient was resuscitated with crystalloids, put on intranasal oxygen, with insertion of a nasogastric tube, and started on intravenous ceftriaxone, metronidazole and omeprazole. The gestational age was 36 weeks, and fetal weight was estimated to be 3000 g based on ultrasound examination. Because she was in labor at presentation, the labor was allowed to continue. Four hours after admission, labor was augmented with oxytocin, and she delivered vaginally. Two hours after delivery, laparotomy and primary closure with omental patch repair was performed by a general surgeon. In terms of the management of the patient, we did not choose immediate laparotomy for both cesarean delivery and repair of the perforated PUD; rather, we preferred allowing vaginal delivery followed by laparotomy for repair of perforated PUD. The reasoning for this choice was: (1) there was no labor abnormality during the follow-up; (2) the patient was multiparous and we assumed that labor would not be abnormal, and (3) vaginal delivery would help reduce the morbidity associated with cesarean section, including contamination of the uterine incision and abdominal wall incisions with abdominal cavity pus, which would increase the risk of surgical site complications, such as infection and dehiscence.

Although perforated PUD is extremely rare during pregnancy, when this occurs, survival of both mother and child is unusual. In their literature review published in 1962, Paul *et al*. described 14 cases of perforated duodenal ulcer in pregnancy in which all 14 mothers lost their lives [9]. In our case, both the mother and the neonate are alive. We searched the English scientific literature and identified and reviewed reported cases of perforated PUD in pregnancy. The maternal and neonatal outcomes are summarized in Table 1.

**Table 1:** Summary of reported cases of perforated peptic ulcer disease in pregnancy and the outcomes

Authors (year)	Maternal age (years)	Gestational age at presentation (weeks)	Clinical presentation	Possible predisposing factor(s)	Diagnostic tool, intraoperative finding, and management	Outcomes
Essilfie *et al*. (2011) [1]	27	38	Recurrent episodes of vomiting, general malaise, back pain, and vague lower abdominal pain that later localized to upper abdomen Significant tenderness in the epigastrium	No significant medical history and was not on medication	Ultrasound showed fluid collection in the right upper quadrant of abdomen Chest X-ray was normal Laparoscopy revealed copious amount of pus and extensive adhesion around the stomach Laparotomy revealed anterior perforation of the second part of duodenum which was repaired and omental patch support created	The delivery was with ventouse and both mother and neonate survived and were discharged on the 7th postoperative day in stable condition
Goel *et al*. (2014) [7]	25	32	Sudden onset severe abdominal pain and nausea but no vomiting Generalized distension with guarding and tenderness, and absent bowel sounds	No known predisposing factor for PUD	Abdominal ultrasound revealed distended bowel loops and mild collection of fluid in peritoneal cavity Possibility of acute pancreatitis was considered and conservatively managed, but no response and laparotomy was decided upon. Intraoperative there was 2.5 L of bile-stained purulent pus in the peritoneal cavity and a 3-cm-long perforation on the first part of the duodenum which was repaired and omental patch support created	Labor started s few hours after laparotomy and a stillborn male neonate weighing 1.8 kg was delivered vaginally The mother was discharged on her 7th postoperative day in improved condition
Gali *et al*. (2011) [10]	16	28	Sudden persistent epigastric pain for 2 days, associated with nausea and vomiting; this occurred during Ramadan fasting period for the Muslims and she was fasting Abdominal examination showed generalized tenderness with guarding, bowel sounds absent	No known predisposing factor for PUD	Air under the diaphragm was detected on the chest X-ray Laparotomy done and revealed 1 L of gastric juice mixed with blood, food debris, and a 1-cm-long perforation on the first part of the duodenum The perforation was closed with omental patch	Labor started 3 days after laparotomy and a living male neonate weighing 1 kg was delivered vaginally, who died 3 days after admission to the special care baby unit The mother developed wound infection which was managed with antibiotics and wound dressing was and discharged 21 days after surgery
Gebremariam *et al*. (2015) [3]	20	28	A 1-day history of supra-umbilical abdominal pain, abdominal distension, and repeated vomiting of coffee ground nature Abdominal examination showed grossly distended tender abdomen	Had history of chronic epigastria discomfort for which she sought no medical advice a or treatment	Initially intestinal obstruction was considered and plain abdominal X-ray was done which showed no any remarkable finding Laparotomy decided upon and intraoperative 2 L of gastrointestinal content and a 1-cm-long anterior wall perforation on the first part of duodenum was found Omental patch was done for the perforation	Postoperatively, induced for severe preeclampsia and delivered vaginally a 1.9-kg dead male neonate The patient was discharged in an improved condition and had no complaints during subsequent follow-up
Erez *et al*. (2004) [11]	27	35	Protracted nausea and vomiting and later development of abdominal pain and tenderness	Maternal bariatric surgery (gastric banding)	The patient was initially diagnosed and treated for a small bowel obstruction but hours later developed acute abdomen and non-reassuring fetal testing. Exploratory laparotomy and cesarean delivery performed. A perforated gastric ulcer was diagnosed and repaired	Emergency cesarean section was done during laparotomy Both mother and neonate survived and discharged home with stable condition
Our case (2022)	35	36	Sudden onset right upper quadrant pain of 7-hours duration that radiated to the back, associated with nausea and vomiting Abdominal examination showed grossly distended, rigid, and diffusely tender abdomen, which showed limited movement with respiration	She had history of intermittent burning type of epigastric pain prior to pregnancy	An upright abdominal X-ray was performed and demonstrated air under diaphragm Ultrasound revealed massive amount of intraperitoneal fluid Laparotomy was done and revealed a copious amount of thin pus in the abdominal cavity, and a 0.5 × 0.5-cm anterior perforation of the first part of the duodenum, which was repaired, and omental patch (Graham’s patch) support was created	Delivered vaginally a 2.9-kg living male neonate with Apgar score of 5 and 8 in the first and fifth minutes, respectively following augmentation with oxytocin The mother and the neonate were discharged 1 week later in stable condition

*PUD* Peptic ulcer disease

## Conclusions

Perforated PUD in pregnancy is rare, which may account for the delay in its diagnosis and management. Therefore, obstetricians should have a high index of suspicion when pregnant mothers present with acute abdomen. Management must be coupled with coordinated care by other disciplinary teams to reduce maternal and fetal morbidity and mortality.

## Data Availability

Data sharing is not applicable to this article as no datasets were generated or analyzed during the current study

## Abbreviations

CT: Computed tomography
FHB: Fetal heart beat
ICU: Intensive care unit
NSAID: Nonsteroidal anti-inflammatory drug
PUD: Peptic ulcer disease
